# Radiation-induced skin injury: a review of pathophysiology, assessment, management, and re-irradiation protocols

**DOI:** 10.3389/fonc.2025.1736717

**Published:** 2026-01-26

**Authors:** Jiao Cheng, Juancong Dong, Yuan Fang, Xiaoquan Zhang, Xuhong Dang

**Affiliations:** 1 Division of Radiology and Environmental Medicine, China Institute for Radiation Protection, Taiyuan, China; 2 Heji Hospital, Changzhi Medical College, Changzhi, China

**Keywords:** radiation dermatitis, radiation-induced skin injury, re-irradiation, skin injury prevention, skin injury treatment

## Abstract

**Background:**

Radiation-induced skin injury (RISI) is a common dose-limiting toxicity of radiotherapy, characterized by erythema, desquamation, fibrosis, atrophy, and ulceration. It results from DNA damage, reactive oxygen species, and dysregulated inflammation.

**Purpose:**

This review synthesizes current knowledge on the mechanisms, assessment tools, and management strategies of RISI, with a focus on emerging therapeutic approaches, particularly in patients requiring re-irradiation.

**Materials and methods:**

A search of PubMed, Embase, and Web of Science for English-language studies from 2020 to 2025 using terms like “radiation dermatitis,” “skin toxicity,” and “stem cell therapy” identified 122 pre-clinical and clinical studies.

**Results:**

Key grading tools for RISI include the Radiation Therapy Oncology Group (RTOG), Common Terminology Criteria for Adverse Events (CTCAE), and Radiation-Induced Skin Reaction Assessment Scale (RISRAS). Conventional treatments like corticosteroids and emollients alleviate symptoms but do not prevent chronic damage. Novel therapies, including mesenchymal stem cells and mitochondrial-targeted antioxidants, show promise in reducing dermal injury and enhancing repair. Re-irradiated patients experience increased severe dermatitis, highlighting the need for better dose-to-skin constraints.

**Conclusion:**

While current management remains mostly palliative, emerging therapies when guided by standardized assessment tools (including the widely used RTOG scale, which remains the clinical gold standard for skin toxicity grading), alongside CTCAE and RISRAS and individualized treatment planning offer hope for reducing acute and long-term skin damage, especially in re-irradiation cases.

## Introduction

Radiation therapy plays a critical role in management of various malignancies particularly cancer (1). The skin, being the outermost and protective barrier of the body, is most affected during both therapeutic and accidental radiation exposure. Skin is the first tissue exposed to external beam radiation along with the basal cell layer and capillaries being particularly susceptible to injury from ionizing radiations (2). Skin injury is frequently reported in radiotherapy, especially in cancer treatment including the breast, head, neck and pelvic region (3). Numerous advanced radiotherapy techniques have been developed rapidly and applied in clinics but exposure of normal tissues is still unavoidable despite increasing accuracy of radiation therapy (4). The incidence of radiation induced skin injuries has elevated gradually, nearly 85%-95% of tumor patients have developed different degrees of skin damage, varying from dermatitis to erythema, depilation, and ulceration (5, 6). Clinical manifestations of RISI include erythema, dry desquamation, moist desquamation, and ulcers.

**Figure 1:**
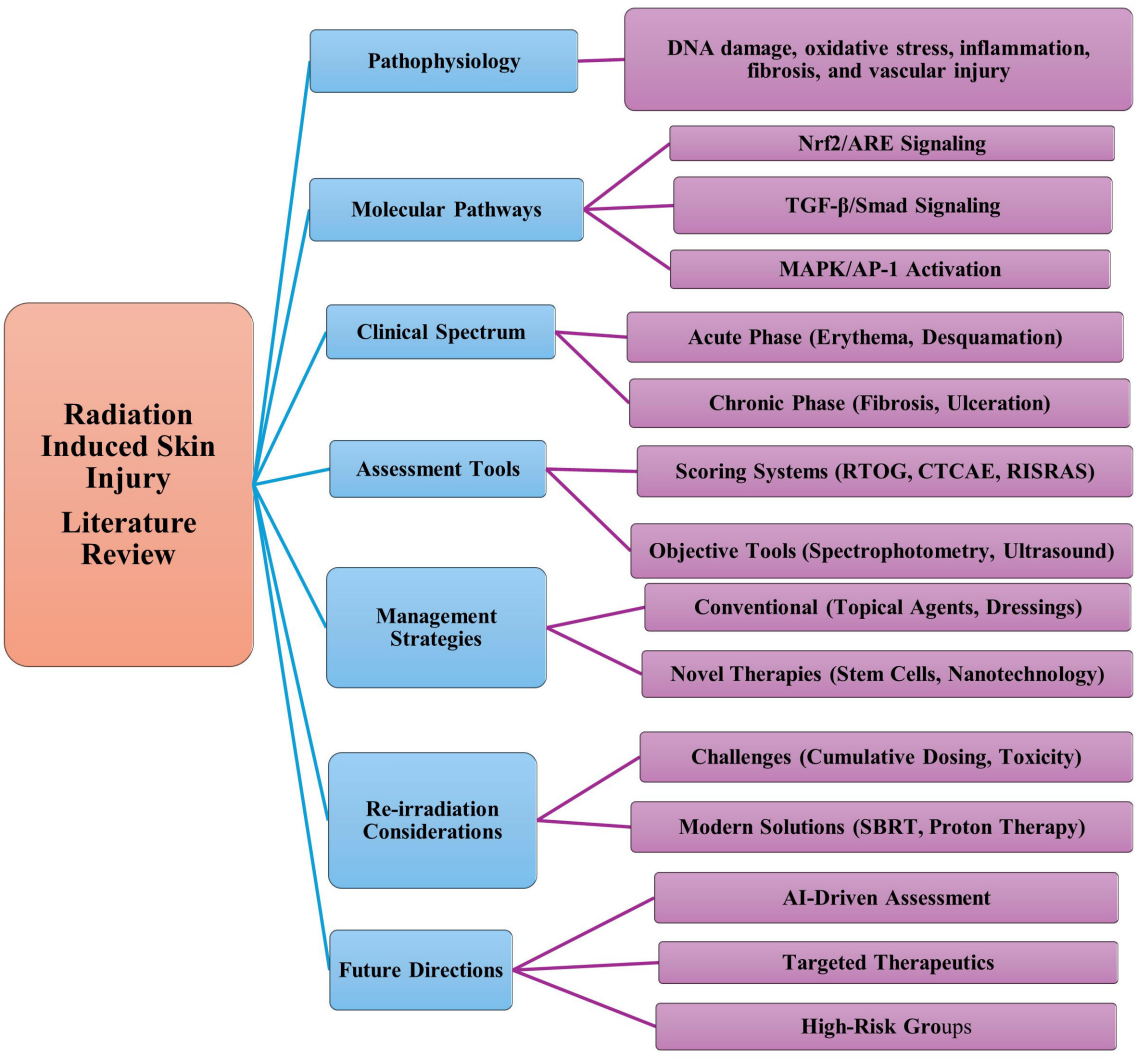
An outline of Radiation Induced Skin Injuries (RISI), highlighting the subjects including pathophysiology, molecular pathways, clinical characteristics, evaluation instruments, and therapeutic approaches. Future direction emphasizes the AI Tools and therapeutic targets. The widely used clinical assessment tool for erythema and skin redness is still referred to as the RTOG scale which is considered as widely used gold standard supported by referenced articles (9, 10).

Radiation induced skin injury is a significant complication, affecting a large proportion of patients undergoing cancer treatment (7). Radiation induced skin injuries not only effect the patient’s quality of life but also slow down healing and decreasing the confidence to fight cancer. The growing prevalence of cancer and adoption of radiation-based therapies has made RISI a serious public health concern. This clinical challenge has spurred significant global research interest, with over a thousand scientific publications on RISI between 2004 and 2023, involving contributors from 66 countries (8). The US alone accounts for 30% of the research on RISI, followed by Japan and China. Current management strategies offer limited potency in addressing symptoms, so there is a need to explore innovative therapeutic approaches and consider the impact of re-irradiation. This review aims to provide a comprehensive overview of RISI, covering its pathogenesis, clinical manifestations, prevention strategies, and potential therapeutic interventions as shown in **Figure 1**.

## Pathogenesis and mechanisms of RISI

**Figure 2:**
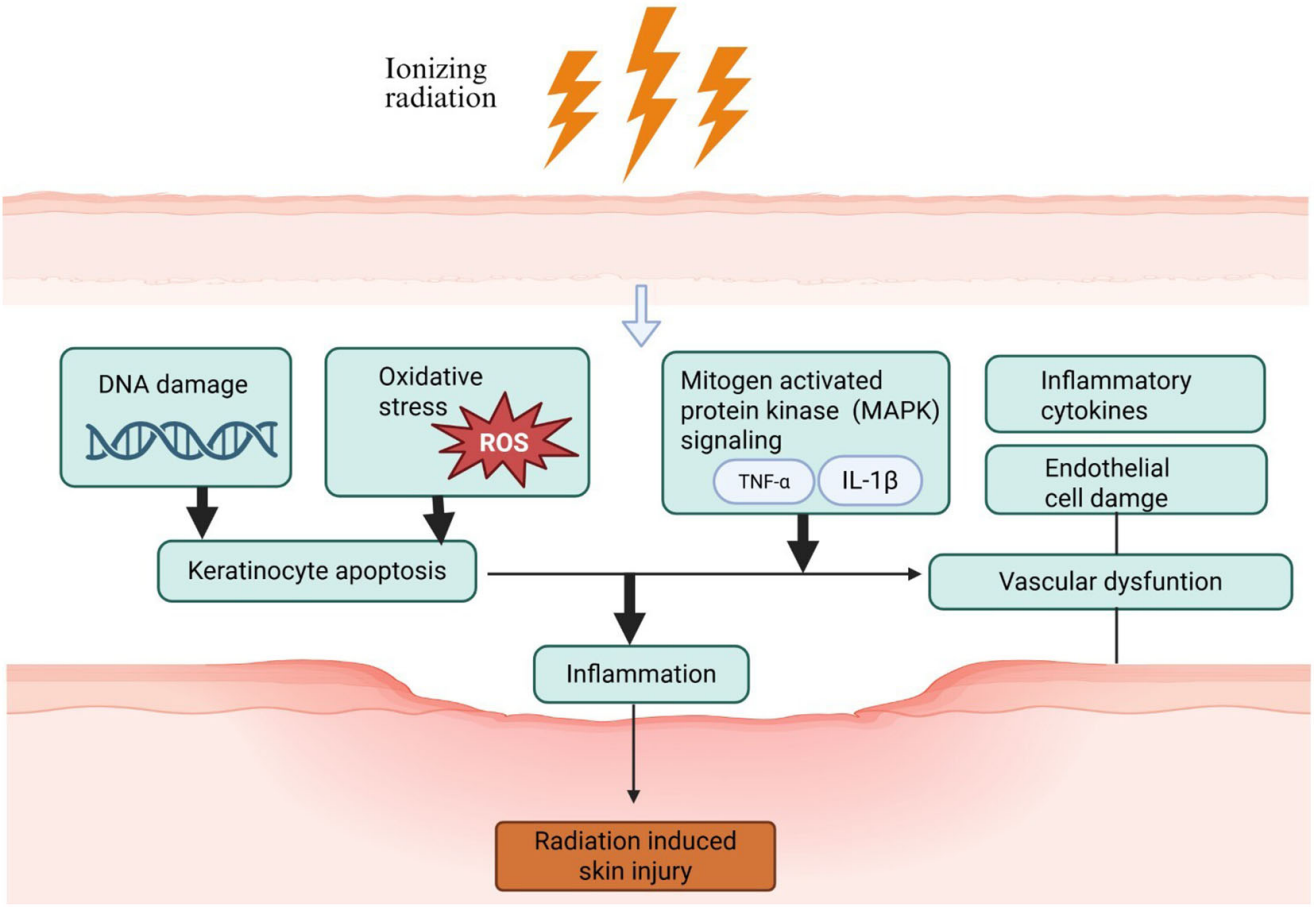
Keratinocyte apoptosis results from oxidative stress (ROS: Reactive Oxygen Species) and DNA damage caused by ionizing radiation. Vascular dysfunction is caused by inflammatory cytokines and endothelial cell damage. These along with MAPK signaling lead to Inflammation and result in Radiation Induced Skin Injury.

The pathogenesis of RISI is multifaceted, involving complex and molecular mechanisms. key factors contributing to radiation induced skin injury includes oxidative stress, inflammation, vascular damage and fibrosis as shown in **Figure 2**.

### Oxidative stress

Ionizing radiation induces the generation of reactive oxygen species (ROS) that leads to oxidative stress (11). Antioxidant enzymes like superoxide dismutase (SOD), catalase (CAT), and glutathione peroxidase (GSH-Px) are activated to counteract the ROS. Excessive ROS production can overburden the oxidative defenses of the skin, causing damage to the biological macromolecules such as DNA, proteins and lipids (12). This oxidative damage triggers a cascade of events that contributes to skin injuries. For instance, UV radiations lead to the production of ROS in the epidermis and dermis that can also activates matrix metalloproteinases (MMPs). It leads to the degradation of the extracellular matrix (ECM), resulting in skin wrinkling.

### Inflammation

Radiation exposure triggers a complex inflammatory response involving various chemicals mediators, cell types and signaling pathways (13). Damage associated molecular patterns (DAMPs) released from damaged cells recruit the immune cells to the site of injury, where they initiate and amplify the inflammatory response (12). These immune cells release inflammatory mediators, such as cytokines, which further exacerbate the inflammatory response (6). Pro-inflammatory cytokines, including IL-6, IL-1β, and TNF-α, play a crucial role in RISI. For instance, activated microglia release pro-inflammatory cytokines such as IL-6 and IL-1β that leads to neuroinflammation.

### Fibrosis

Excessive fibrotic remodeling can occur in the later stages of RISI. The release of growth factors and continuous inflammation can activate fibroblasts that leads to increased production of extracellular matrix (ECM) such as collagen (14). This causes remodeling of the (ECM) and the development of fibrosis, which can impair tissue function leading to chronic radiation induced skin injuries.

### Vascular damage

Radiation directly impacts the vasculature by endothelial cell injury and apoptosis. Vascular damage is a critical component in pathogenesis of RISI by causing senescence (15). Microvascular endothelial cells are particularly sensitive to ionizing radiation and radiation induced apoptosis. Exposure to ionizing radiations causes the release of oxygen radicals and proteases, resulting in leukocyte dysfunction, loss of endothelial barrier function that eventually leads to organ damage (16). In addition, radiotherapy can accelerate atherosclerosis, resulting in vascular events.

## Molecular mechanisms of RISI

**Figure 3:**
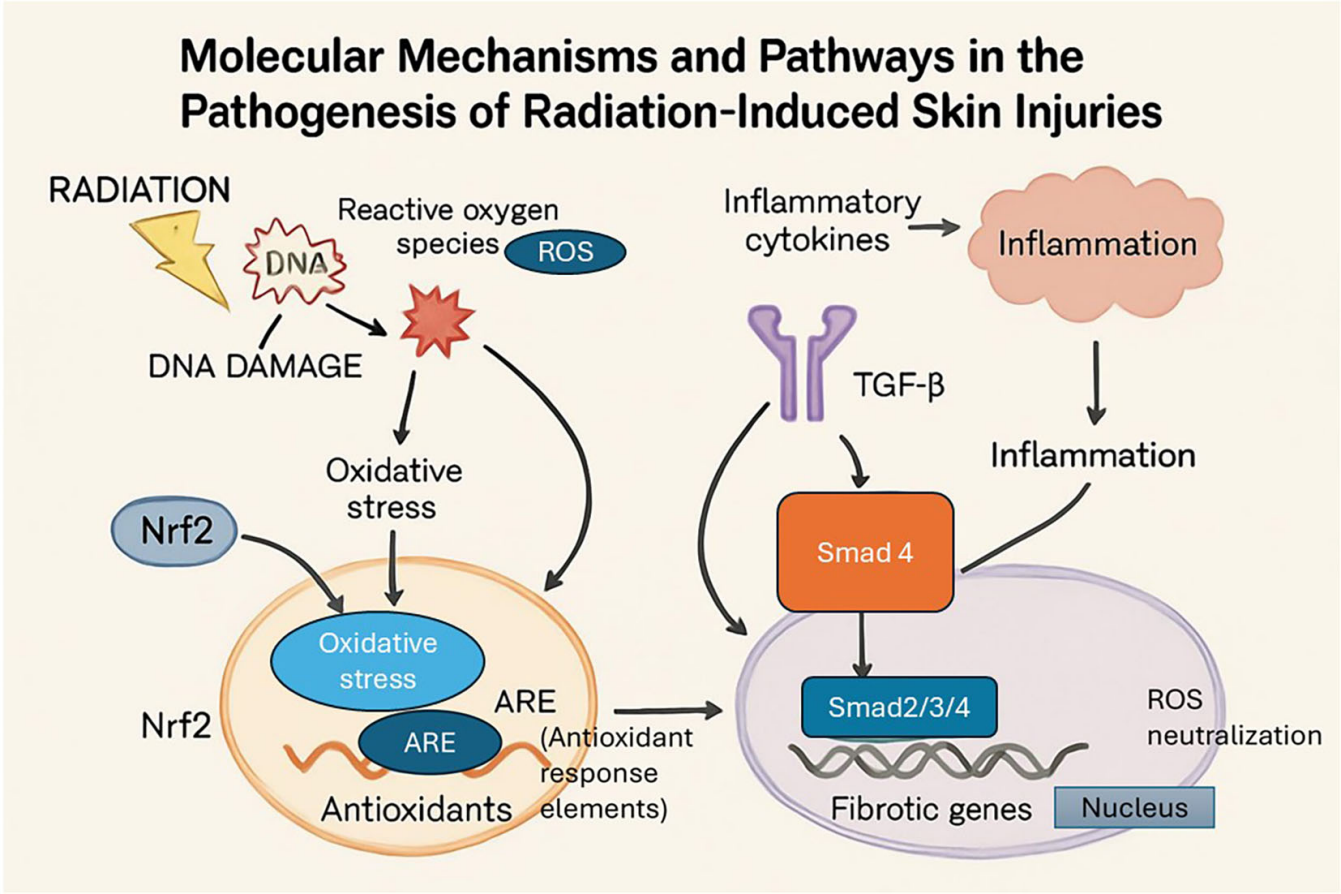
Molecular pathways that contribute to RISI focusing on inflammation and oxidative stress. Radiation activates Nrf2 and TGF-β/Smad signaling, causes DNA damage, and produces ROS. These pathways stimulate inflammation and tissue damage by controlling fibrotic gene expression and antioxidant defences.

Various molecular pathways are implicated in pathogenesis of radiation induced skin injury as shown in **Figure 3** (11).

### Nrf2 pathway

The nuclear factor erythroid 2-related factor 2 (Nrf2) is the primary regulator of the cellular antioxidant response (17). Activation of the Nrf2 pathway results in the expression of antioxidant and cytoprotective genes that helps to alleviate the oxidative stress, thereby offering protection against radiation induced injuries as described in **Figure 3**. Experimental studies have shown that Nrf2 deficient mice are more susceptible to RISI, underscoring the critical protective function of this pathway (4). Glycocalyx sialic acid has been found to modulate Nrf2 pathway in response to fluid shear stress in human endothelial cells which suggests a broader regulatory mechanism for Nrf2 activity (18).

### TGF-β/Smad signaling

The transforming growth factor-beta plays a crucial role in tissue fibrosis. TGF-beta promotes the differentiation of fibroblasts into contractile myofibroblasts and stimulate the overproduction of collagen, fibronectin, and other ECM components (19). In case of radiation injury, persistent TGF-β signaling contributes to the major hallmarks of late-stage RISI such as chronic inflammation, excessive ECM deposition and dermal fibrosis. This pathway has shown promise in preclinical model, where inhibition of TGF-β and downstream Smad signaling to reduce fibrosis in models of tissue injury and improve skin healing outcomes (20).

### Inflammatory cytokines

**Figure 4:**
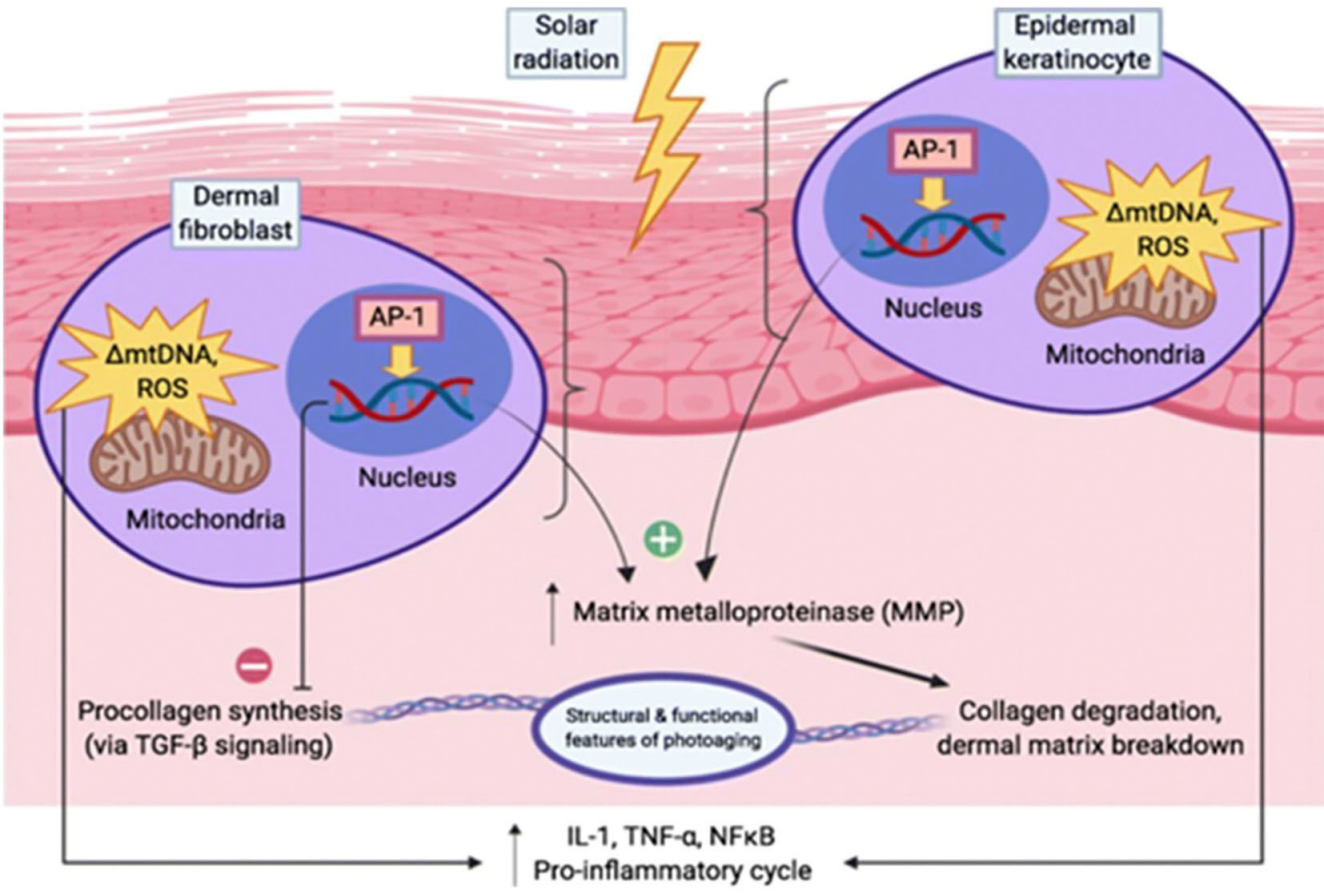
Solar radiation induces oxidative stress and DNA damage in skin cells. Activates MMPs, disrupts Collagen Synthesis, and promotes Dermal Matrix Degradation. Triggers Pro-Inflammatory Signaling (IL-1, TNF-α, NFκB) accelerating Photoaging.

Pro inflammatory cytokines such as IL-1, TNF-α, and NF-κb are involved in sustaining inflammatory response within the skin (1). When skin cells such as keratinocytes, fibroblasts and immune cell are exposed to external stressors such as ultraviolet and ionizing radiations, they become activated and secrete these cytokines (21). These cytokines further contribute to skin damage and photoaging. The IR-induced ROS activate a critical signaling cascade, the mitogen activated protein kinase (MAPK) pathway. Activation of MAPK results in phosphorylation and release of transcription factors like ATF-2 and AP-1 (4). The transcription factor AP-1 binds to the promotor region of matrix metalloproteinases (MMP-1) which results in collagen fiber breakdown and results in wrinkled skin as seen in photoaging as shown in **Figure 4**.

## Clinical assessment tools and assessors

Radiation-induced skin injury is typically assessed and graded by the treating radiation oncology team. To effectively assess the degree of radiation induced skin injury (RISI), the RTOG, CTCAE and RISRAS scales are used (22). Reliable screening of the RISI is vital for swift action and interpretation of the results. Assessments can vary, considering every scale has different standards to evaluate RISI. The choice of scale depends upon the type of toxicity and the particular condition.

### RTOG (radiation therapy oncology group)

One of the most widely used grading system for Radiation-Induced adverse reactions including skin toxicities is RTOG scale (9). It uses clinical symptoms such as erythema, edema and desquamation to qualitatively analyze the intensity of RISI. It is a clinician-based numerical classification from grade 0(no change) to grade 4(ulceration, necrosis). It is a basic and helpful system however it is not sensitive enough to observe any minute changes or the patient described symptoms.

### CTCAE (common terminology criteria for adverse events)

In clinical trials, the clinicians typically use the National Cancer Institute (NCI)’s Common Terminology Criteria for Adverse Events to report the Adverse Events (AEs). The CTCAE also uses the clinician’s observations to grade the severity of symptoms and has five grades. The CTCAE is commonly used to grade the intensity of Acute Radiation Dermatitis (ARD) however, despite decades of clinical use, it has not been validated. The National Cancer Institute (NCI) designed the Patient-Reported Outcomes version of the Common Terminology Criteria for Adverse Events (PRO-CTCAE) to gauge clinical adverse events (AEs) directly from the patients participating in cancer clinical trials (22). By recording the patient’s viewpoint on the frequency, intensity, and interruption of symptoms to everyday life, it is meant to add to the commonly used Common Terminology Criteria for Adverse Events (CTCAE), which are reported by clinicians (23).

### RISRAS (radiation-induced skin reaction assessment scale)

The RISRAS is a comprehensive grading scale (scoring between 0 to 36) integrating both the clinician-based and patient reported outcomes, especially devised to evaluate the skin symptoms related with radiation dermatitis (24). It enables the patients to report the intensity and implications of their skin reactions making it a patient-centered approach on RISI as detailed in **Table 1**.

**Table 1:** Comparison of the RTOG, CTCAE and RISRAS scales used to assess the severity of radiation induced skin injury.

Parameters for the evaluation of the scales	RTOG (Radiation Therapy Oncology Group)	CTCAE (Common Terminology Criteria for Adverse Events)	RISRAS (Radiation-Induced Skin Reaction Assessment Scale)
Developed by	Committee for Radiation Therapy Studies (CRTS)	National Cancer Institute (NCI)	Rae Noble-Adams
Basis of the Assessment	Clinician-based	Clinician-based	Clinician-based and Patient reported
Scale Range	Grade 1-4	Grade 1-5	Scores 0-36 Researcher’s Component (total scores between 0 to 24) Patients Component (total scores between 0 to 12)
Aim of the Grading	General assessment of radiation toxicity (25)	All adverse events in a clinical trial (26)	Radiation-induced skin reactions (24)
Sensitivity towards Patient’s Experience	Low	Intermediate	High
Estimated Factors	Erythema, desquamation, ulceration	Skin reaction severity, functional impact, treatment needs	Erythema, desquamation (clinician) + pain, itching, burning (patient)
Benefits	Easy to use and prevalent in radiation oncology	Comprehensive scoring that is consistent between trials	Thorough; attention to minor modifications; incorporates patient symptoms
Drawbacks	Subjective and void of information about symptoms	More detailed but still information id sieved from clinician.	Less frequently employed outside of research settings; demands dual assessment
Usage	Standard clinical evaluation	Surveillance of adverse reactions and clinical trials	Research and evaluation of care focused on patients
Acceptability	Routinely employed in radiotherapy	Universal in Clinical studies of Oncology	Provides more detailed skin response studies but less standardized

## Role of objective tools

Although the subjective scales are valuable but provide the qualitative data. To support them, the objective tools such as spectrophotometry and ultrasound are used as they offer an even more thorough evaluation of RISI.

Spectrophotometry is a technique used to objectively measure skin color by quantifying the intensity of reflected light at specific wavelengths. This method makes it possible to identify subclinical inflammation early on and offers repeatable data for monitoring treatment response or progression. Spectral assessment techniques have been shown to quantify radiation-therapy–induced skin erythema with high accuracy, supporting their role in early, objective evaluation of cutaneous responses (27). Pilot studies using hyperspectral imaging have demonstrated its effectiveness in detecting spatial and temporal changes in radiotherapy-induced skin erythema (28). Noninvasive spectral monitoring methods have further confirmed the ability of optical technologies to track inflammation progression during radiation therapy (29). Common symptoms associated with RISI such as erythema and hyperpigmentation can be successfully evaluated by observing the changes in skin pigmentation. The skin’s spectrophotometric values match the EORTC/RTOG-Common Toxicity Criteria. To compare and measure the melanin levels in the skin, a non-invasive and objective method called Diffuse Reflectance Spectroscopy (DRS) is used. Balneo PUVA therapy treated vitiligo skin is assessed using this method to measure the melanin levels. Serum Magnesium ions level are also evaluated using spectrophotometry, it provides both benefits and drawbacks compared to atomic absorption spectrophotometry and inductively coupled plasma–optical emission spectrophotometry (30).

Ultrasound uses high-frequency sound waves to visualize the subcutaneous tissues and is a non-invasive imaging technique (31). When evaluating skin thickness, edema, and subcutaneous fibrosis, high-frequency ultrasound (20 MHz or more) is helpful, particularly in cases of chronic or advanced RISI. High-Frequency Ultrasonography (HFUS) is used for the examination and diagnosis of cutaneous melanocytic tumors (32). In order to lower the extent of the biopsy and enhance cosmetic results, ultrasound can assist in evaluating the margins of lesions. Additionally, it can distinguish between benign and malignant tumors. Critical anatomical information that cannot be inferred by palpation, dermoscopy, confocal microscopy, magnetic resonance imaging, or PET-CT techniques is provided by ultrasound (31).

## Current clinical management and treatment of RISI

**Figure 5:**
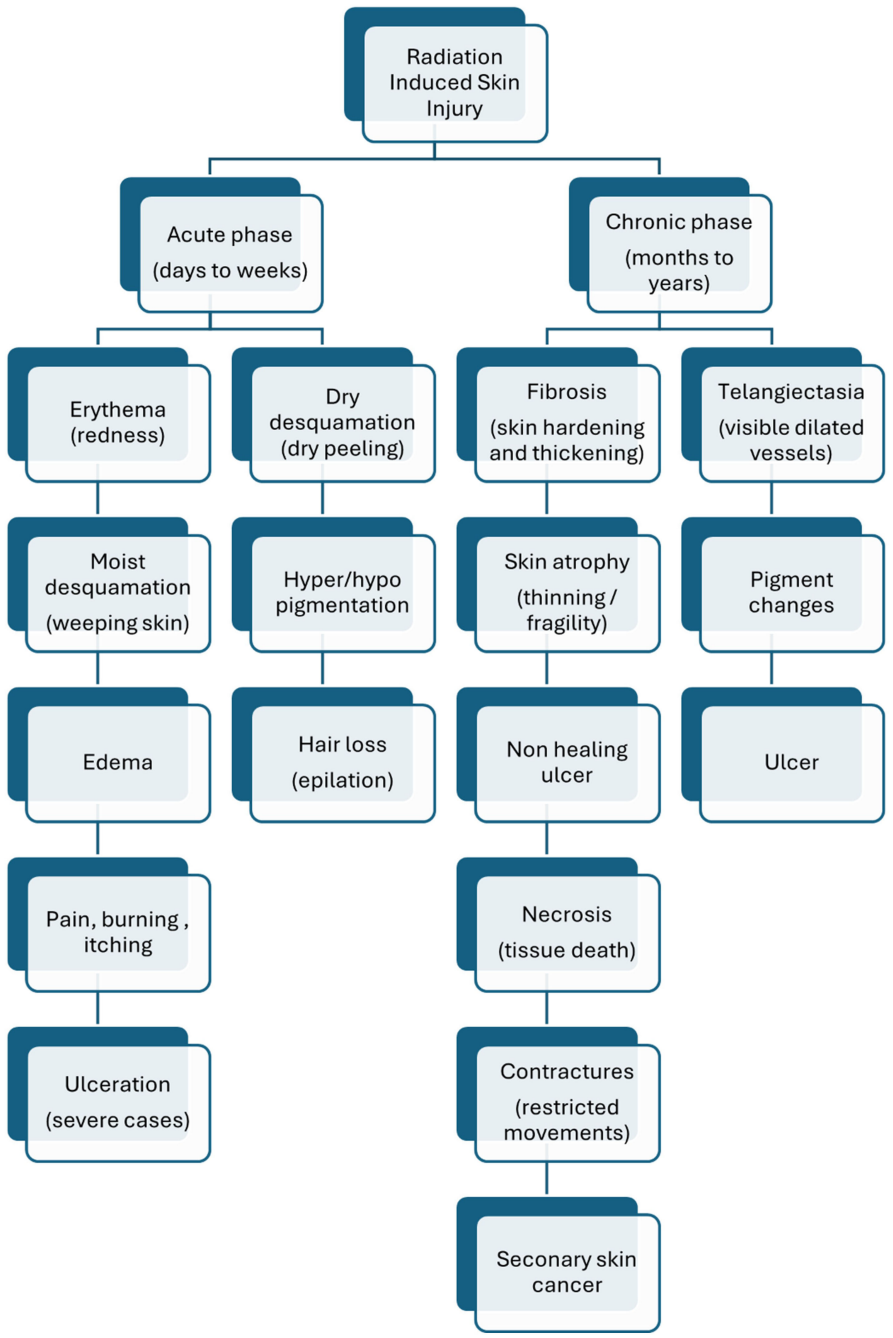
Spectrum of radiation induced skin injuries, Timeline and clinical features from acute-phase erythema and desquamation to chronic effects like fibrosis, telangiectasia, and secondary malignancies.

Clinical indications of RISI are often graded based on severity, with common grading scales including those by the Common Terminology Criteria for Adverse Events (CTCAE) as described in **Figure 5**.

Grade 1: Faint erythema
Grade 2: Moderate to brisk Erythema
Grade 3: Confluent moist desquamation or pitting edema
Grade 4: Skin ulceration, necrosis, full thickness skin loss
Grade 5: Death

## Step-by-step management algorithm

No single gold standard exists for the treatment of RISI, but a comprehensive approach incorporating prevention and management is crucial.

### Acute phase management

These are the effects observed after or during therapy session. Acute radiation dermatitis is observed in all patients undergoing radiotherapy to some extent. Effective management of side-effects involves phase-specific strategy that revolves around prevention, symptom control and avoiding treatment interruptions.

### Prophylaxis

During preventive measures, it is best to educate patients about their symptoms and expected side-effects so they can manage themselves without panicking. As the sensitive skin can be easily irritated so it is better to avoid irritants like perfumes, harsh soap, and wear loose clothing. Use lukewarm water and natural cleansers. Stay away from chemical-containing, irritants and strong products as these may flare-up the skin (33). Moisturizers and emollients should be used in daily routine to keep the skin hydrated and barrier functional, hyaluronic acid creams showed promising improvement in skin integration and hydration (34). If there is any inflammation on skin, topical corticosteroids like hydrocortisone can be applied to reduce inflammation and pruritus (optional). With many advancements in science, experimental agents (novel radioprotectors) are also available (e.g., Erb-IL10 fusion proteins, ROS scavengers) that can help in reducing inflammation or alleviating certain side-effects of radiotherapy or chemotherapy (34).

### Acute phase management

#### (Symptomatic relief and grade-specific treatment options)

##### Grade 1

Grade 1 is characterized by faint erythema (redness on skin) (1). It is best to continue the skin care regimen and moisturization as indicated in prophylaxis. To reduce itchiness and redness, topical corticosteroids can be incorporated. Other supportive creams that are soothing can also be used for this grade to lessen skin irritation and erythema such as aloe vera, calendula (35).

##### Grade 2

This grade is characterized by moderate erythema, which means that the skin has more redness than usual (1). Keep the wound clean and avoid friction. As skin is already aggravated and sensitive thus use non-adherent dressings to protect and maintain moisture balance. If the wound becomes infected, use antiseptics or antibiotic creams. Oral analgesics can also be taken to reduce the pain if needed (36).

##### Grade 3

Pitting edema and confluent skin desquamation are the main characteristics of grade 3. Advanced dressings with hydrogels or silver-impregnated dressings are employed to ensure enough hydration as skin is very sensitive during this phase (37). If needed, systemic antibiotics can be taken to combat secondary infections. In order to manage severe pain opioid analgesics can be taken.

##### Grade 4

At this stage, skin ulceration and necrosis can be detected on patient so wound debridement can be considered to limit the spread (remove narcotic tissue). Specialized dressings or reconstructive surgery should be used because full thickness skin loss can be seen along-with necrosis. Close monitoring is advised for sepsis management (11). Monitoring through prediction tools like detecting early skin temperature change and predicting ARD grades using V50 is also in practice (38).

### Chronic phase management

Chronic RISI can affect the patient’s quality of life drastically as the body weakens severely at this stage, involving persistent erythema, fibrosis, hyperpigmentation, telangiectasis, and ulceration on a long-term basis.

#### Assessment

Evaluation of chronic radiation-induced skin injury. Biopsy is conducted to exclude malignancy or related conditions (39). Life quality is assessed through social interaction, self-image, and mobility.

#### Fibrosis/scarring

Physical therapy or massage is indicated at this stage to improve skin elasticity. Topical agents like retinoids, vitamin E can be used but there is little evidence supporting their benefits in this phase (34). Surgical options can be considered such as fat grafting (40).

#### Chronic ulceration

Removal of the necrotic tissue and employing advanced therapies like skin substitutes, growth factors, and hyperbaric oxygen may be beneficial for non-healing ulcers (41). The ability of glycopeptide hydrogels to modulate chronic inflammation and accelerate healing of wounds is being investigated in many studies (36). Stem cell therapy is also a promising area for treating chronic phase of radiation induced injury.

#### Hyperpigmentation/telangiectasia

For vascular lesions or telangiectasia (these are small visible blood vessels that appears on skin. i.e. broken capillaries), laser therapy can be used. Topical hydroquinone or retinoid can also be integrated for lightening of hyperpigmentation and discoloration on skin. These treatments can reduce the side-effects to some extent.

#### Pain

**Figure 6:**
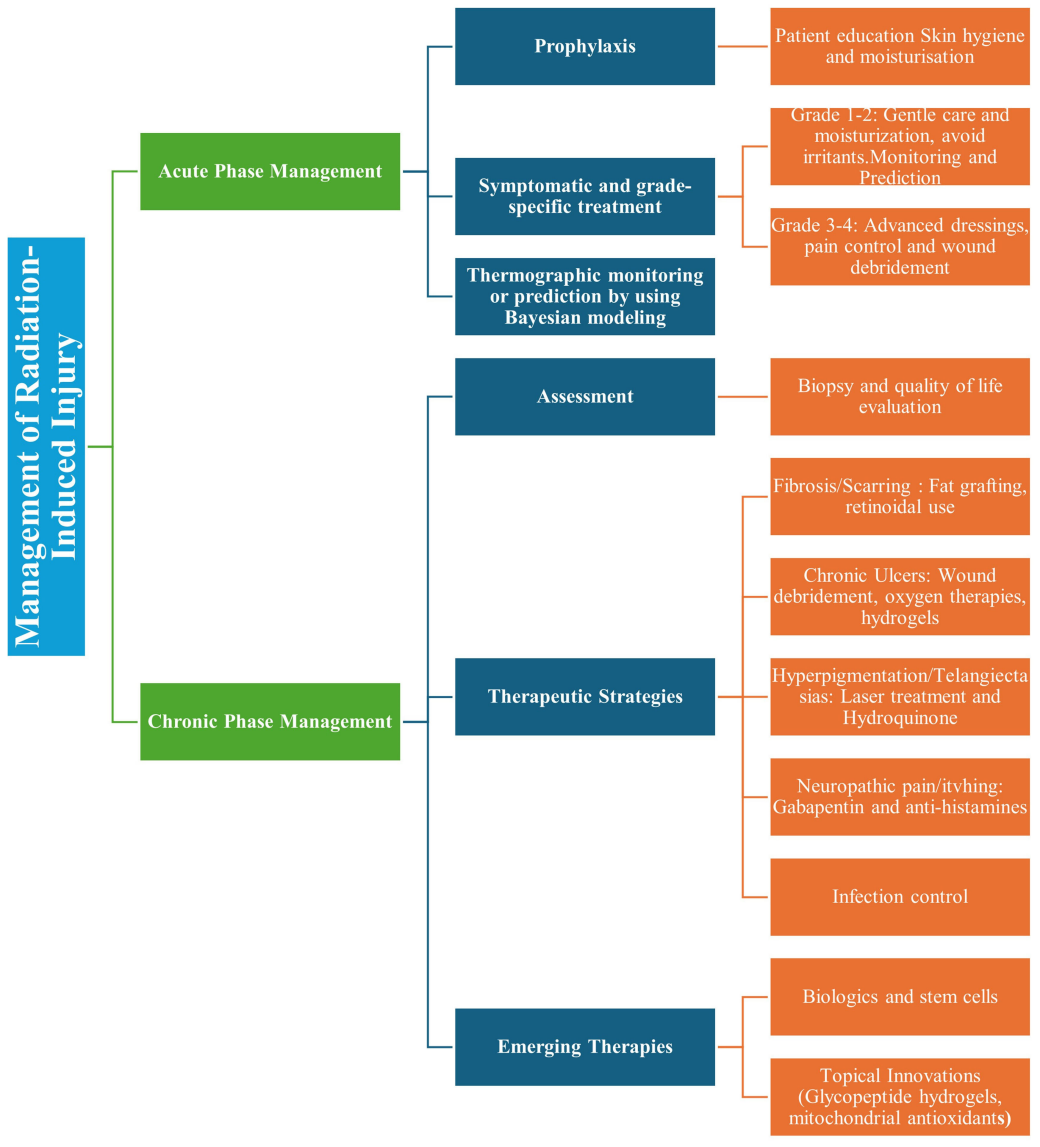
Prophylaxis and management of radiation induced skin injuries covering acute-phase symptom control (graded care, monitoring) and chronic-phase interventions (fibrosis treatment, advanced therapies). Highlights include patient education, wound care, and emerging options like biologics and hydrogels.

In chronic phase, patient can experience sharp and uncontrollable pain so, neuropathic pain agents such as gabapentin and pregabalin can be given to them for some relief (3). For symptomatic relief of itching, antihistamines or anti-itch creams can used. A summarized management strategy can be seen below in **Figure 6** (42).

## Evidence-based interventions

Improved and evidence-based comprehensive strategies have replaced the subjective approaches in the management of radiation induced skin injury. Treatments differ depending upon the state and extent of the RISI and include pharmacological, dressing-based, and device-assisted therapies.

## Topical agents for RISI

### Hyaluronic acid

Hyaluronic Acid based formulations in gel or cream forms, are an effective wound dressing and proven for the localized treatment of cancer due to their hydrophilicity, biocompatibility, potential targeting property, and highly adaptable structure for modulated functionalities (43). As a fundamental component of the skin’s extracellular matrix, hyaluronic acid plays a vital role in key processes including tissue regeneration, angiogenesis, and inflammation (44). Hyaluronic acid released from the membrane stimulates wound healing by promoting re-epithelialization.

### Kangfuxin

The Radiation Induced Skin Injuries are successfully managed using kangfuxin solution. Derived from an American Cockroach named Periplaneta americana, it has anti-ulcerative and wound healing properties. Kangfuxin has demonstrated potential in the treatment of oral mucositis and dermatitis caused by radiations. It reduces the intensity of desquamation and speeds up the healing process in patients undergoing radiation therapy (45–47).

### Topical corticosteroids

Topical Corticosteroids are used for the treatment of Radiation Induced Skin Injuries (48). For atopic dermatitis, they are the first line of treatment (49). The use of TCs is potentially beneficial for managing radiation dermatitis by analyzing the randomized control trials and prospective studies on the use of TCs in radiation dermatitis (48). Topical corticosteroids efficiently alleviate the inflammation in a variety of skin disorders. Systemic Corticosteroids are also available but should be used carefully because of their severe adverse effects (50).

## Advanced dressings for RISI

- Hydrogels are a significant improvement in the field of wound healing. Hydrogels can absorb exudates, retain a moist environment and facilitate tissue regeneration, and are biocompatible. They provide an optimal environment to promote the healing of skin and re-epithelialization (51). Wound Closure is achieved using the hydrogels as they maintain a moist environment necessary for the cellular migration, tissue regeneration and proliferation (51).Hydrogels acts as a physical barrier preventing the exogenous contamination of the wounds during healing process and can be tailored according to the wound size to provide complete coverage. Nanoparticles are also incorporated into the hydrogels to prevent microbial infections and to maximize the competency of the dressing (52).
- Silver-Impregnated Dressings are silver-containing dressings combined with hydrogels commonly used for managing Immunosuppression Induced Skin Ulcerations (IISU) (53). They are effective in controlling infections in immunocompromised patients and in palliative settings. Silver nanoparticles (AgNPs) are antibacterial and proven for wound healing but face challenges such as high cost (54).
- Alginate Dressings are made of alginate membranes impregnated with hyaluronic acid and Silver Nanoparticles (AgNPs) and are used to treat chronic non-healing wounds as it supports and accelerates the wound healing process.

## Device-based therapies for RISI

- Low Level Laser Therapy uses low-intensity, red or infrared light to stimulate the activity of mitochondria, reduce inflammation, collagen synthesis and other cellular processes thus promotes wound healing (55). When administered prophylactically or therapeutically, clinical trials in individuals with head and neck cancer have shown decreased discomfort, rapid healing, and a reduced number of grade ≥2 skin responses.
- Fractional Radiofrequency (FRF) is a device based therapy is not used as a primary treatment for the RISI, however it is a physical stimulation tool promoting blood circulation and stimulating new cell growth (56) so it can be a beneficial approach for managing Radiations Induced Skin Injuries. Further studies should be conducted to specifically evaluate FRF for RISI (57).

## Novel therapeutics and targeted therapies

- Stem Cell Therapy has shown potential by promoting tissue regrowth and recovery in radiation damaged skin. The stem cells differentiate into specific cell types and release paracrine factors that reduce inflammation and enhance the healing process (58). For improved wound healing, hydrogel-based multipurpose dressings can incorporate stem cell treatment and magnetothermally sensitive medication administration (59).
- Mitochondria-Targeting Conjugates are radio protective hemicyanine (HCY) small compounds that are coupled with the plasminogen inhibitor tranexamic acid (TA) which target the mitochondria. By inhibiting plasminogen, these HCY-TA conjugates combine anti-inflammatory, antioxidant, and wound-healing qualities to treat RISI (59).
- Nanotechnology-Based Drug Delivery increase therapeutic accuracy and lower the systemic adverse effects, making it a feasible approach to treat RISI (6). Protein absorption, tumor cell growth, tumor cell adhesion, tumor angiogenesis, and cancer metastasis all can be suppressed with integrative strategies that use HA (60). Targeting particular RISI-affected cells or tissues is possible with Nano carrier development (61). Various Nano-sized carriers like liposomes, polymeric nanoparticles, dendrimers, and micelles are used.

## Re-irradiation therapy and cumulative dosing thresholds

Ionizing radiation stimulates a complicated series of chemical and cellular processes that lead to radiation-induced skin injury. The skin is the first line of defense and barrier against foreign objects, and it is the primary target for radiation exposure, whether therapeutic or unintentional. RISI damages cells directly (3) and can induce reactive oxygen species (ROS) that ultimately activate cell death via necrosis and apoptosis (62). The risk of RISI is seen to be as high as 100% and more severe in head and neck cancers, while often lower in other types of cancers (63). Re-irradiation is often considered for patients with recurring malignancies in formerly treated areas. For example, it is an advanced strategy, particularly in head and neck cancers (41). Re-irradiation is being evaluated more frequently as it helps in the reduction of local tumors, but it also increases the risk of severe tissue harm, including RISI.

## Cumulative dosing thresholds

The ability of normal tissues to withstand re-irradiation is an important concern. Normal tissues can heal some damage with the passage of time, but the exact process is unknown. Generally, cumulative doses over 100 Gy can increase the risk of serious consequences considerably. Although there is no commonly recognized safe cumulative dosage threshold for all tissue types. Tissues that have been exposed to persistent radiation-induced damage become more vulnerable to additional radiation exposures. This brings immediate attention to optimizing and formulating strategies for re-irradiation volumes and recommended dosages that benefit therapeutically more efficiently with lower toxicity (64).

For example, the cumulative incidence of radiation-related gliomas rises substantially with time, especially 10 years post-radiation (65). The re-irradiation chain is comprised of appropriate evaluation, contouring, dose prescription for the maximal therapeutic ratio, plan creation and adjustment, which is followed by a diligent follow-up for acute and late toxicities (66).

## Reirradiation and stereotactic body radiation therapy

Stereotactic body radiation therapy (SBRT) limits the exposure to the adjacent healthy tissues as it specifically targets malignancies by administering high doses of radiation in a few fractions. Thus, it is being administered more frequently for re-irradiation in a variety of tumors, such as head and neck (67), spinal (68), and gastrointestinal cancers, due to its relatively safe profile in re-irradiation therapy.

Careful dose planning is very important for SBRT in re-irradiation settings to avoid going over the tolerance of normal tissue. For example, in recurrent head and neck cancer, when organ sparring techniques are implemented, SBRT re-irradiation has demonstrated an acceptable toxicity profile and enhanced life quality outcomes.

## Proton therapy and re-irradiation

Due to the Bragg peak feature, proton treatment provides more efficient dose distribution than photon therapy. This allows for accurate dose deposition within the tumor while protecting neighboring healthy tissues, resulting in a potentially decreased cumulative dose to previously irradiated normal tissue (69–71).

Proton therapy reduces overlap with previous radiation doses, and it may improve the side effect profile for re-irradiation in persistent gliomas (72).

### Prevention protocols during SBRT/proton therapy

Preventive measures are crucial to reduce toxicity and to ensure therapeutic effectiveness during stereotactic body radiation therapy (SBRT) and proton therapy. These protocols are customized to the tumor site and patient-specific characteristics, including mobility management, dosage restrictions, imaging guidance, and toxicity mitigation strategies. Current practices from the literature are given below.

### Patient selection and risk assessment

The foundation of SBRT safety lies in careful patient selection. A thorough evaluation of the tumor characteristics, patient comorbidities, and the proximity of the target volume to critical organs at risk (OARs) is carried out for this purpose. For example, SBRT is already an established treatment for early-stage, inoperable lung cancer but its role in high-risk prostate cancer is still under investigation (73).

## Image-guided radiotherapy and motion management

### Dosimetric considerations

The goal is to maximize the preservation of the surrounding healthy tissues while administering a high, conformal dosage to the tumor (72, 73). This includes inverse planning techniques. In proton SBRT, fewer beam angles can reduce low-dose radiation to normal tissues significantly compared to photon SBRT (74).

### Quality assurance and safety protocols

Safety protocols and quality assurance were much needed as these treatments involve high doses and short courses, giving rise to many challenges (75, 76). As compared to traditional radiation therapy, SBRT is associated with a higher risk of errors, ultimately requiring a need of robust safety protocols. The protocols include careful equipment commissioning, specialized staff training, and specific workflow differences in comparison to conventional radiotherapy (77).

**Figure 7:**
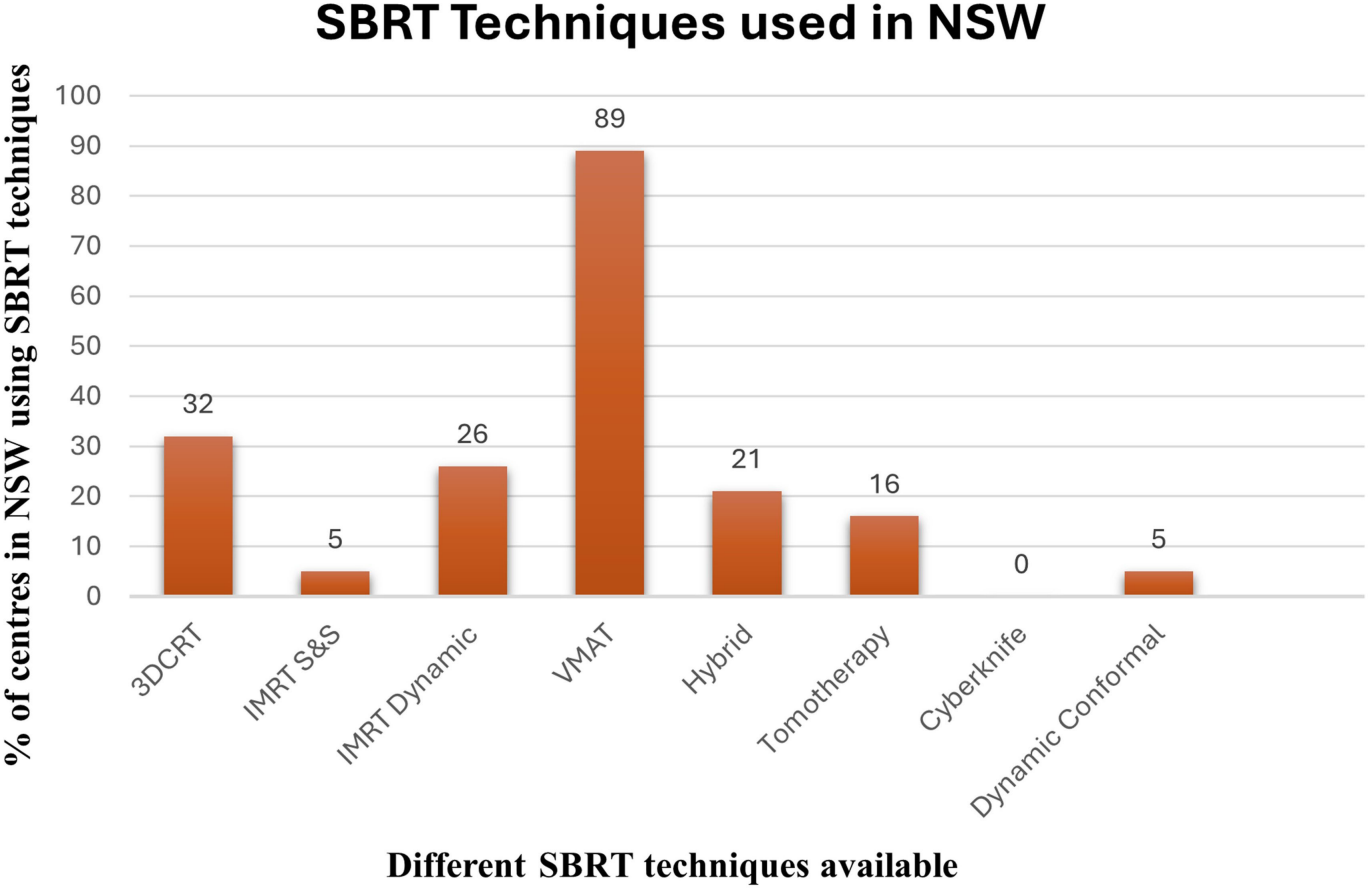
Bar chart visualization of SRBT techniques adopted in NSW reflecting the preferences of different centres and availability of technology across the NSW centres.

Advancements in technology have encouraged the use of SBRT worldwide. Techniques like Volumetric Modulated Arc Therapy (VMAT), 3D Conformal Radiation Therapy (3DCRT), Intensity Modulated Radiation Therapy (IMRT), etc. are used to attain conformal dose delivery (75). The bar chart shown in the **Figure 7** illustrates the percentage of centers using different Stereotactic Ablative Body Radiation Therapy (SABR) techniques (75). In the study of Mackonis, there is comparison of different SBRT techniques available and being used in different centers in NSW (New South Wales).

### Toxicity (acute and late toxicities)

Prostate studies revealed that indeed SBRT is effective, but it can lead to acute gastrointestinal and genitourinary toxicities, which generally increase in the weeks following treatment and then stabilize (76). Genitourinary (GU) Toxicity Prevention: In an effort to reduce acute urinary retention, alpha-blockers can be prescribed (e.g., tamsulosin) during prostate SBRT (78).

### High-risk groups

Certain patient groups are at higher risk of severe radiation-induced skin injury (RISI).

#### Diabetes

Diabetic patients are already associated with complications like diabetic foot ulcer due to poor circulation and nerve loss and impaired wound healing due to damaged microvasculature. So, they are prone to more serious RISI, leading to discomfort and reduced quality of life. For such patients, nanomedicine approaches that target diabetes-induced damages (oxidative stress, inflammation, poor angiogenesis) may improve the outcomes after radiation therapy (79).

#### Collagen disorders

The extracellular matrix of skin contains collagen, which is essential for skin integrity and repair. Patients with degraded collagen may have reduced ability of the skin to heal or withstand injury in response to radiation exposure.

Skin damage raises significant morbidity risks in the curative treatment of malignancies, even though skin tissue is not dose-limited by radiation, particularly when radiation and chemotherapy are used together. Subcutaneous fibrosis and morbidity are the most severe late side effects in the spectrum of radiation-induced damage. Studies showed that UV radiation can reduce collagen concentration and certain approaches aiming to maintain collagen and its integrity can mitigate damage (80).

## Patients undergoing simultaneous systematic therapies

Certain targeted therapies or medicines (like bevacizumab) can make tissues more sensitive to radiation, causing serious side effects, including gastrointestinal fistulas or perforations in cervical cancer patients receiving pelvic radiation therapy (81).

## Future directions

Future research on radiation induced skin injury (RISI) should concentrate on addressing gap in the current understanding of pathogenesis of the disease, improving clinical assessment tools, exploring innovative management strategies, and optimizing re-irradiation protocols (41). Development of targeted therapies and predictive biomarkers is crucial (66). Novel technologies such as AI-driven assessment tools and precision medicine should be considered to enhance patient specific management techniques. Investigating skin microbial barrier and its alterations may provide insight to the pathophysiology of RISI (82). Furthermore, understanding the impact of radiation on hyaluronan degradation and its underlying mechanisms may reveal new therapeutic approach. Identification of molecular targets for RISI treatment based on the Nrf2 pathway, TGF-β/Smad signaling, and inflammatory cytokines can offer a novel therapeutic strategy (83). Precision medicine approaches involve use of molecular profiling to guide treatment decisions and personalize therapy (84). Further investigation into the potential of stem cells and their derived exosomes for RISI treatment and clinical trials of combination therapies, prophylactic strategies, and optimized wound care protocols are critical to establish evidence-based standards of care.

In conclusion, radiation induced skin injury remain a complex and multifaceted complication that affects a large proportion of patients. Despite advancements in understanding the molecular pathogenesis of RISI, significant gaps remain in translating molecular insights. Management of RISI requires a comprehensive and interdisciplinary approach. Incorporating robust clinical assessment tools and introducing innovative therapeutics are crucial steps toward minimizing the burden of RISI. Moreover, the increasing necessity of re-irradiation approach highlights the need for vigilant planning and protective measure. Ongoing research on pathogenesis of RISI and interdisciplinary collaborations will be compelling for transforming scientific advances into clinical solutions, with the aim of enhancing outcomes and quality of life for patients suffering from RISI.
